# Meropenem/Vaborbactam and Cefiderocol as Combination or Monotherapy to Treat Multi-Drug Resistant Gram-Negative Infections: A Regional Cross-Sectional Survey from Piedmont Infectious Disease Unit Network (PIDUN)

**DOI:** 10.3390/jfb13040174

**Published:** 2022-10-03

**Authors:** Tommaso Lupia, Silvia Corcione, Nour Shbaklo, Giorgia Montrucchio, Ilaria De Benedetto, Valentina Fornari, Roberta Bosio, Barbara Rizzello, Simone Mornese Pinna, Luca Brazzi, Francesco Giuseppe De Rosa

**Affiliations:** 1 Unit of Infectious Diseases, Cardinal Massaia, 14100 Asti, Italy; 2 Department of Medical Sciences, Infectious Diseases, University of Turin, 10126 Turin, Italy; 3 School of Medicine, Tufts University, Boston, MA 02111, USA; 4 Department of Surgical Sciences, University of Turin, 10126 Turin, Italy; 5 Department of Anaesthesia, Critical Care and Emergency, Città Della Salute e Della Scienza Hospital, Corso Dogliotti 14, 10126 Turin, Italy

**Keywords:** survey, meropenem/vaborbactam, cefiderocol, intensive care unit, stewardship, multi-drug resistant

## Abstract

Meropenem/vaborbactam (MV) and cefiderocol were recently approved by the Food and Drug Administration and European Medicines Agency and are among the most promising antibacterial in treatment regimens against multi-drug resistant (MDR) gram-negative bacilli. A survey with close-ended questions was proposed to infectious disease (ID) and intensive care unit (ICU) physicians of Piedmont and Valle d’Aosta Region’s hospitals. The aim was to collect data about habits and prescriptions of cefiderocol and MV. Twenty-three physicians (11 ID specialists and 12 anesthesiologists) in 13 Italian hospitals took part in the survey. Both cefiderocol and MV were mostly used as target therapy after a previous treatment failure and after ID specialist consult. The most frequent MDR pathogen in hospitals was *Klebsiella pneumoniae* carbapenemase-producing bacteria (KPC), followed by *P. aeruginosa* and *A. baumannii*. MDRs were more frequently isolated in ICU. In conclusion, cefiderocol was used in empiric regimens when *A. baumannii* was suspected, while MV was more used in suspect of KPC. MV and cefiderocol can be the first option in empiric treatment for critically ill patients in settings with high risk of MDR. The treatment should then be followed by rapid de-escalation when microbiological results are available.

## 1. Introduction

The appearance and spread of new mechanisms of bacterial resistance to antibiotics are serious health problems. Among the most difficult resistance mechanisms to treat are the production of extended-spectrum beta-lactamases (ESBL), ampicillinase C (AmpC), carbapenemases (CP), Metallo-beta-lactamases (MBL) and oxacillinase (OXA) enzymes released by Gram-negative bacteria [1]. However, exciting developments of new antimicrobial drugs, especially anti-Gram-negative antibiotics with a spectrum covering multidrug-resistant microorganisms (MDROs), have characterised research innovations in the last several years [2,3,4]. Although, the rapid evolution of antimicrobial regimens and options against MDROs requires infectious diseases specialists to constantly update their knowledge and engage in continuous research to make the best use of new molecules and insert them correctly into antimicrobial stewardship programmes (ASPs) [5].

Recently, meropenem/vaborbactam and cefiderocol were approved by the Food and Drug Administration and European Medicines Agency and are among the most promising molecules in treatment regimens against MDR gram-negative bacilli (GNB) [6,7,8,9].

Meropenem/vaborbactam is a novel carbapenem-boronic acid β-lactamase inhibitor formulation that presents antimicrobial activity against classes A and C β-lactamase-producing *Enterobacterales*, especially those producing the ESBL, KPC and AmpC determinants [2,3]. The efficacy, tolerability, and safety of meropenem/vaborbactam for the treatment of complicated urinary tract infections (cUTIs) and acute pyelonephritis have been investigated in phase 3 non-inferiority trial, TANGO I [10]. Thereafter, a second randomised, open-label trial, TANGO II, investigated patients with cUTIs, hospital and ventilator-acquired pneumonia, bacteremia or complicated intra-abdominal infections (cIAIs) due to known or suspected carbapenem-resistant *Enterobacteriaceae* infection [11].

Second, cefiderocol is a combination of a catechol-type siderophore and a cephalosporin core with side chains similar to cefepime and ceftazidime [2,4]; its structure and mechanism of action confer enhanced stability against hydrolysis by many β-lactamases, such as CTX-M, and carbapenemases, such as KPC, NDM, VIM, IMP, OXA-23, OXA-48-like, OXA-51-like and OXA-58 [2,4]. Cefiderocol has a broad antibacterial spectrum against a variety of aerobic bacteria, including *Enterobacterales*, *Acinetobacter* spp., *Pseudomonas* spp., *Burkholderia* spp. and *Stenotrophomonas maltophilia* [2,4]. In cefiderocol studies, there appears to be a trend towards poorer outcomes compared to standard antibiotic therapy if the bacterium is not multi-drug resistant [2,4,12,13]. In CREDIBLE-CR, cefiderocol was associated with favourable microbiological outcomes compared to the best available therapy when it came to cUTIs [12]. Moreover, in APEKS-NP a randomised, double-blind, phase 3, non-inferiority investigation, cefiderocol was found to be non-inferior to high-dose extended-infusion meropenem in patients with Gram-negative nosocomial pneumonia [13]. Few data are available so far about the best combination therapy for the management of MDR infections and the use of monotherapy and its clinical impact is still debated.

This regional survey aimed to describe the clinical prescribing habits surrounding the use of MV and cefiderocol in the ICU setting and its potential role in ASPs.

## 2. Materials and Methods

The study involved a cross-sectional internet-based questionnaire on prescribing habits for MV and cefiderocol. The questionnaire was designed with close-ended questions and distributed using the SurveyMonkey platform (San Mateo, CA, USA). Informed consent was waived due to the nature of the study (survey). We requested information on participants’ specialty and hospital name, size, and type. The survey was mostly intended for infectious disease specialists and intensive care physicians which provides advice on antibiotic treatments in the Piedmont and Valle d’Aosta Region (Italy) and members of the Piedmont Infectious Diseases Unit Network (PIDUN).

The questionnaire was developed by four primary investigators and pre-tested by two other authors for clarity and technical functionality.

We asked respondents to reply by describing the most common actual practices at their hospitals. A maximum of two participants from each hospital was included.

Cefiderocol and meropenem/vaborbactam used in this survey did not include compassionate use or expanded access use.

### 2.1. Survey Administration

One investigator submitted a proposal to join the questionnaire through e-mail. After a positive response, an invitation was sent by the survey coordinator. Participants were able to access the questionnaire multiple times to allow modification and completion upon their convenience. The survey was voluntary, with no incentives offered to participants (other than being listed as collaborators). Participants had six months (from November 2021 to May 2022) to access the questionnaire. Recruitment criteria for physicians were registered as infectious disease or ICU specialist in his own country, minimum experience of 2 years as a specialist, able to communicate through the internet and members of PIDUN

### 2.2. Response Rates

Response rates were calculated as the number of clinicians from whom an answer was recorded. Information on hospital names was used to screen for duplicate entries, but all data were subsequently anonymized for the analyses.

### 2.3. Statistical Analysis

Both completed and partially completed questionnaires were analyzed using the number of completed responses per item as the denominator.

## 3. Results

Twenty-three clinicians joined the questionnaire through e-mail. The clinicians involved were distributed across 13 Italian hospitals located in the Piedmont and Valle d’Aosta regions. The centers were located in Alessandria, Aosta, Asti, Cuneo, Verduno, Novara, Torino, Vercelli, Moncalieri, Pinerolo and Rivoli.

Among the considered hospitals, 56.52% (12) had between 200 and 500 beds, 39.13% (8) had more than 500 beds, and 4.35% (1) had 100–200 beds; all had at least one ICU, with slightly more than half (52.4%) having more than one; in most of the cases, secondary ICUs specialized in neurosurgery and cardio surgery (Table 1).

The participants were equally distributed between infectious disease specialists and anaesthesiologists (52.17% and 47.83%, respectively). In most of the centres involved, an infectious disease specialist was physically present 7 days a week (14; 60.87%). In three cases, the infectious diseases specialist was either present locally 5 days per week or available to go on-site after a phone consult (13.04% and 17.39%, respectively). In 82.62% of the centres involved, both cefiderocol and meropenem/vaborbactam were available; in one case, only the latter was available (Table 2).

Both cefiderocol and meropenem/vaborbactam were used as targeted therapies in most cases (95.65% and 91.3%, respectively), after either microbiologic or clinical failures in the previous regimens (73.91% and 69.57% of the cases, respectively). Most of the prescriptions for cefiderocol or meropenem/vaborbactam followed an infectious disease specialist consult (17; 82.61%). Both cefiderocol and meropenem/vaborbactam were administered as 3 h infusion, or sometimes as continuous infusion as described in Table 2.

In 16 cases (69.57%), the survey participants reported that the hospital had available molecular resistance testing for multi-drug resistant (MDR) bacteria, and four of them reported the need to send the samples to a nearby center for testing.

The most used test for cefiderocol or meropenem/vaborbactam resistance was the E-test (43.48% and 43.48%, respectively); the less frequently used tests were rapid antimicrobial susceptibility testing (RAST) and the microdilution broth method.

The interviewed clinicians were asked about which GNB MDR was most frequent in their hospital and to describe the percentage (<5%, 5–15%, 15–30% or >30%) for each Enterobacterales MDR during the last 12 months; they reported that *Klebsiella pneumoniae* KPC+ was identified as the most frequent MDR pathogen in 56.52% of the hospitals, followed by *P. aeruginosa* MDR (26.09%) and *A. baumannii* MDR (17.39%) as showed in Table 3.

In particular, MDR Enterobacterales were reported as isolated in 5–15% and 15–30% of the samples in the majority of cases (34.78% respectively); MDR *A. baumannii* was reported to be observed in <5% of the samples in half of the cases (47.83%; 11) and in 5–15% of the samples in 34.78% of the cases (8); MDR *P. aeruginosa* was reported as less than 5% of the samples or 5–15% (34.78, respectively (Table 3).

All MDR pathogens investigated were most frequently isolated in the ICU. MDR Enterobacterales were isolated in the ICU in 56.62% of the cases. MDR *A. baumannii* was the most frequently found in the ICU (73.91%) and less frequently in surgical and medical wards (4.35% and 8.7% of the cases, respectively) (Table 3).

In 87% of cases, cefiderocol was used as empiric or targeted therapy when *A. baumannii* was the suspected pathogen, followed by MDR *P. aeruginosa* (21.74%) and *K. pneumoniae* VIM+/NDM+ (13.04%) were chosen for empiric therapy.

The most frequent MDR pathogen suspected when starting meropenem/vaborbactam either as the empirical or target therapy was K. Pneumoniae KPC+ (82.62% of the cases), and other CP Enterobacterales (17.39% and 17.39%, respectively) for both empirical and target therapies.

The most used pharmacological association with treating MDR *A. baumannii* infections involving cefiderocol was fosfomycin (66.7%), followed by colistin (52.4%) and ampicillin/sulbactam (42.9%) (Table 4). Fewer clinicians reported rifampicin or tigecycline associations (2 and 5 cases, respectively). However, the most reported association with cefiderocol for MDR *P. aeruginosa* infection treatment was fosfomycin (66.7), followed by aminoglycosides and colistin (38.1% and 33.3%, respectively). Fosfomycin plus cefiderocol was also the association of choice to treat Escherichia coli or *K. pneumoniae* NDM+ or VDM+ (38.1%) and *K. pneumoniae* KPC+ or OXA-48+ (61.9%). Despite that, in some cases, cefiderocol was used as monotherapy as well. 

Regarding meropenem/vaborbactam, it was combined most frequently with fosfomycin (56.52) or aminoglycosides (39.13) and chosen as monotherapy in 26.09% of infections caused by *K. pneumoniae* KPC+.

## 4. Discussion

Rello et al. recently conducted a survey of infectious disease specialists, microbiologists, and intensivists to identify differences in their perceptions of MDROs and their management in the ICU [14]. The survey found differences between the priorities of infectious disease specialists and those of intensivists regarding organisms, infection control practices and educational priorities, highlighting the distinct approaches and points of view of infectious disease and ICU specialists while also stressing the need for close cooperation [14].

Our survey explored infectious disease consultants’ and intensive care physicians’ practices of prescribing meropenem/vaborbactam and cefiderocol for the management of MDROs, with a particular interest in ICU settings in the Piedmont and Valle d’Aosta regions of Italy in a six-month period in 2021–2022. In their first months of availability, meropenem/vaborbactam and cefiderocol were used mainly as targeted therapy directly on MDROs and, in most cases, after a different previous regimen had clinically or microbiologically failed to cure the infection. 

The debate about the most valuable option to use these new drugs, is still open, especially regarding the choice between monotherapy and combination therapy. Recommendations are not univocal for MDROs: clinical setting, pathogen isolation, infection source and disease’s severity assessment should guide the prescription [15,16,17,18,19,20,21,22].

Respondents from 13 hospitals in 10 cities homogeneously represented the Piedmont territory and Valle d’Aosta region. Our results indicate that the respondents worked mainly in hospitals with from 200 to 500 beds. All the included hospitals had at least one ICU, and about half relied on at least one resuscitation specialist (Table 1). The respondents had a high median average work experience and were equally distributed between infectious disease specialists and anaesthesiologists (Table 1).

All the participating hospitals had the opportunity to contact the infectious disease consultant directly by telephone; an infectious diseases specialist was physically present locally in only 71.4% of the hospitals between five and seven days per week. 

In this survey, ETEST^®^ was the most used test to prove meropenem/vaborbactam resistance. In about 16 h, it can determine the MIC level for antimicrobial susceptibility of Gram-negative aerobic bacteria; it provides clinically relevant information for appropriate antimicrobial therapy decisions and reduces the development of drug-resistant bacteria [23]; this is despite the fact that EUCAST prescribes broth microdilution as the reference method for the antimicrobial susceptibility testing of rapidly growing aerobic bacteria, except in the cases of mecillinam and fosfomycin, for which agar dilution is the reference method [24] At this moment, there is no approved ETEST^®^ or other gradient strip for cefiderocol except for *P. aeruginosa* [25]. In addition, cefiderocol exhibits some peculiarities determining MIC by broth microdilution, as it must be performed in an iron-depleted Mueller-Hinton broth, and specific reading instructions must be followed [24,25]. For these reasons, microbiological susceptibility testing may be improved by the wider adoption of broth microdilution determinations for these new molecules [24,25].

Moreover, in 14.3% of centers, molecular resistance testing on MDROs was sent outside the hospital, possibly creating delays in diagnosis and screening. In the hospitals included in this survey, delays in MDRO diagnosis may have also been prolonged by the occasional need to send the microbiological samples out of the hospital. Fast microbiology quickly detects the presence of pathogens and clinically relevant determinants of antibiotic resistance, offering the potential for early administration of antibiotics [26]. Employing these new diagnostic methods will allow a more accurate etiological diagnosis, the discovery of new pathogens and, globally, better patient management [26]. Implementing these methods requires a significant investment, the training of clinicians and laboratory staff and the availability of equipment and reagents [26].

Both cefiderocol and meropenem/vaborbactam were infused in 3 h in most cases (71.4% and 81% of the cases, respectively). Meropenem/vaborbactam is prescribed at a dose of 2000/2000 mg every 8 h as a 3 h infusion and cefiderocol is administered IV at a dose of 2000 mg every 8 h, as a 3 h infusion [6,7,8,9]. Multiple daily dosing coupled with prolonged infusion (i.e., extended, or continuous infusion) may represent the best approach to maximize the time-dependent antimicrobial activity of beta-lactam antibiotics [27]. Among the novel beta-lactams or cephalosporins, only meropenem–vaborbactam, cefiderocol (extended infusion in 3 h) and ceftazidime/avibactam (extended infusion in 2 h or prolonged infusion in more than 3 h) were evaluated using this rationale [17,27]. To our knowledge, no continuous infusion reports are published regarding meropenem/vaborbactam and cefiderocol.

The microbiological ecology in these centers involved in the survey varies according to the hospital and territory. Despite that, in the hospitals involved in this survey, *Klebsiella pneumoniae* KPC-producer was identified as the most frequent MDR pathogen (57.1%); this finding is in line with a recent study published by Bianco and colleagues reporting microbiological data on 1242 non-duplicate *Enterobacterales* clinical strains, collected during the period 2019–2021 in Piedmont, Italy [28]. In their study, Bianco et al. showed a high prevalence of KPC-producers’ strains (n = 1034, 83,2%). Moreover, the most frequent common *Enterobacterales* was *Klebsiella pneumoniae* (87.6%), and 93% of *K. pneumoniae* strains harbored the KPC enzyme [28].

In our survey, MDR *Enterobacterales* were reported as isolated between 5 to 30% of the samples in most hospitals (N = 14, 66.7%); this survey was completed during a pandemic period and also the microbiological data reported, are to be read with this bias. Recently, Shbaklo et al. reported in our region a significant increase in all MDR infections during the COVID-19 pandemic [29]. In particular, compared to the pre-COVID period, *K. pneumoniae* KPC+ (14% vs. 23%), carbapenem-resistant *A. baumannii* (1.5% vs. 5%) and carbapenem-resistant *P. aeruginosa* (3% vs. 4%) have all risen [29].

Respondents chose meropenem/vaborbactam mainly when *K.pneumoniae*-CP was suspected; this appears in line with what is conditionally recommended by European Society of Clinical Microbiology and Infectious Diseases (ESCMID) guidelines to treat severe infections due to CRE, while the use of an old antibiotic is still proposed as the first choice to treat non-severe infections. Despite to date, the ESCMID guidelines do not recommend combination therapy for CRE infections (strong recommendation, low evidence), we observe that the majority of respondents use meropenem/vaborbactam in association with other drugs [30]. In a multicentric retrospective study, ceftazidime-avibactam monotherapy, ceftazidime-avibactam combination therapy and meropenem-vaborbactam monotherapy have been compared in patients with severe CRE infections, including bacteremia mainly caused by *K. pneumoniae*-CP and no significant difference in clinical success and mortality rate was observed even though patients in the meropenem-vaborbactam arm received combination therapy more rarely than in the ceftazidime-avibactam arm [31]. Similarly, Tumbarello et al. [18] in a multicentric cohort of KPC-*K. pneumoniae* bloodstream infections and low respiratory tract infections showed safe and successful use of meropenem/vaborbactam monotherapy and meropenem/vaborbactam combination therapy with colistin or fosfomycin in recurrent cases. Similarly, to this, in meropenem/vaborbactam association use, the majority of our respondents prefer fosfomycin followed by aminoglycosides and polymixin, both for KPC-*K. pneumoniae* and *Pseudomonas aeruginosa* MDR. Respondents chose cefiderocol mainly when MDR *A. baumannii* was suspected. In a recent retrospective study, the use of cefiderocol monotherapy in MDR *A. baumannii* infections showed no differences in the all-cause mortality rate compared to the colistin-treated group [32]. Similar results were obtained when a population including *Stenotrophomonas maltophilia* and MBL producing *Enterobacterales* was analysed [33]. A small number of respondents agreed with the use of cefiderocol monotherapy in treating MDR *A. baumannii* or *Pseudomonas aeruginosa* MDR, while a slightly higher proportion of them agree with the use of cefiderocol monotherapy in the treatment of CRE sustained infections including those producing MBL. Interestingly, also in this case fosfomycin was the more frequently proposed travel companion for all analyzed pathogens, including NDM/VIM, exceeding aztreonam by large.

Despite we aimed at describing the clinical prescribing habits, the different availability of meropenem/vaborbactam and cefiderocol in the enrolled centers, as well as the different lab disposability in terms of molecular resistance testing, may represent limitations of this study, affecting the single respondent’s unconditioned reporting. Moreover, the use of close-ended questions, may have driven respondents to simplification biases, providing answers fitting into pre-conceived categories.

In our opinion, meropenem/vaborbactam and cefiderocol may be the first option as an empirically therapy in critically ill patients with known risk factors for MDR, or known colonization by MDROs or suspected carbabapenem resistant GNB infections, as well as in endemic setting for MDR (<25% MDR in the hospital setting) followed by a rapid antibiotic de-escalation and targeting isolates with preliminary microbiological results; these molecules can be used either in combination or monotherapy, according to the availability of microbiological results. Cefiderocol or meropenem/vaborbactam based monotherapy could be considered as a targeted therapy centered on fast microbiology findings and local epidemiology data, whereas combination therapy may represent an appropriate choice in critically ill patients with pending microbiology results.

## 5. Conclusions

In conclusion, our survey described a wide heterogeneity approach for the management of GNB MDR infections, either between infectious disease or ICU specialists. Both cefiderocol and meropenem/vaborbactam were mostly used as target therapy after a previous treatment failure and after an infectious disease specialist consult. The most frequent MDR pathogen in hospitals was KPC, followed by *Pseudomonas aeruginosa* and *Acinetobacter baumannii*. MDRs were more frequently isolated in ICU. Cefiderocol was used in empiric regimens when *A. baumannii* was suspected, while meropenem/vaborbactam was more used in the suspect of KPC. Meropenem/vaborbactam and cefiderocol can be the first option in empiric treatment for critically ill patients in settings with high risk of MDR. The treatment should then be followed by rapid reassessment when microbiological results are available. 

## Figures and Tables

**Table 1:** Main characteristics of the respondents and the centers involved.

**Number of Beds in Your Hospital**	**<100**	**100–200**	**200–500**	**>500**
Total (23/23)	0 (0)	1 (4.35)	12 (56.52)	9 (39.13)
**Role Played in the Hospital**	**ID**	**Intensivist/Reanimator**		
Total (23/23)	12 (52.17%)	11 (47.83%)		
**ID Consultant**	**In-Hospital 7-days**	**In-Hospital 5-days**	**In-Hospital 2-days**	**On-Call**
Total (23/23)	14 (60.87)	3 (13.04)	2 (8.7)	4 (17.39)
**Intensivist/Reanimator**	**General**	**Specialized**		
Total (11/23)	10 (92.31)	1 (7.69; Neuro)		
**Years’ Service**	**Median (IQR)**			
Total (22/23)	17 (2–35)			

Abbreviations: ID: infectious disease; IQR: interquartile range.

**Table 2:** Availability and Habits of using for Cefiderocol and Meropenem/Vaborbactam.

	Median (IQR)		
Total (19/23)	10 (1–30)		
Number of patients treated M/V per Respondant	Median (IQR)		
Total (19/23)	4 (1–40)		
Time of availability of Cefiderocol in your center	Median (IQR)		
	8 (1–24)		
Time of availability of M/V in your center	Median (IQR)		
	8 (1–18)		
**Modality of Infusion**	**Cefiderocol**	**M/V**	
Continuous	2 (8.7)	1 (4.35)	
>3 h	5 (21.74)	4 (17.39)	
Up to 3 h	16 (69.57)	18 (78.26)	
**Cefiderocol choice (more than one response)**	**Empirical**	**Targeted**	
Total (23/23)	2 (8.7)	22 (95.65)	
**M/V choice (more than one response)**	**Empirical**	**Targeted**	
Total (23/23)	5 (21.74)	21 (91.30)	
Cefiderocol choice (more than one response)	Clinical Failure	Microbiological failure	Both
Total (19/23)	2 (8.7)	0 (0)	17 (73.91)
**M/V choice (more than one response)**			
Total (20/23)	3 (13.04)	3 (13.04)	16 (69.57)
**M/V or Cefiderocol choice after ID consultation (more than one)**	**Yes**	**No**	
Total (23/23)	19 (82.61)	4 (17.39)	

Abbreviations: M/V: meropenem/vaborbactam.

**Table 3:** Rates and types of MDR Gram-negative bacilli from the centers involved in this survey.

Rates of MDR *Enterobacterales* from Clinical Isolates	<5%	5–15%	15–30%	>30%
Total (23/23)	4 (17.39)	8 (34.78)	8 (34.78)	3 (13.04)
Rates of Carbapenem-resistant *A. baumannii* from Clinical Isolates	<5%	5–15%	15–30%	>30%
Total (23/23)	11 (47.83)	8 (34.78)	3 (13.04)	1 (4.35)
Rates of Carbapenem-resistant *P. aeruginosa* from Clinical Isolates	<5%	5–15%	15–30%	>30%
Total (23/23)	8 (34.78)	8 (34.78)	7 (30.43)	0 (0)
Higher rate of MDR Enterobacterales	Medical Wards	Surgical Wards	ICU	Not Known
Total (23/23)	8 (34.78)	0 (0)	13 (56.52)	2 (8.7)
Higher rate of Carbapenem-resistant *A. baumannii*	Medical Wards	Surgical Wards	ICU	Not Known
Total (23/23)	2 (8.7)	1 (4.35)	17 (73.91)	3 (13.04)
Higher rate of Carbapenem-resistant *P. aeruginosa*	Medical Wards	Surgical Wards	ICU	Not Known
Total (23/23)	6 (26.09)	0 (0)	14 (60.87)	3 (13.04)
More frequent MDR Enterobacterales from Clinical Isolates	*K. pneumoniae* KPC+	*A. baumannii* MDR	*P. aeruginosa*	*S. maltophilia*
Total (23/23)	13 (56.52%)	4 (17.39)	6 (26.09)	0 (0)

Abbreviations: ICU: intensive care units.

**Table 4:** Use of Meropenem/Vaborbactam and Cefiderocol as empirical and targeted therapies.

**Preferred Empirical Use of Cefiderocol**	**Isolates**	**N (%)**
Total (23/23)	Kp VIM or NDM +	3 (13.04)
	*P. aeruginosa* MDR	5 (21.74)
	*A. baumannii* MDR	20 (86.96)
	*S. maltophilia*	1 (4.35)
	Other Enterobacterales	1 (4.35)
**Preferred empirical use of M/V**	**Isolates**	**N (%)**
Total (23/23)	Kp KPC+	19 (82.61)
	*P. aeruginosa* MDR	4 (17.39)
	Other Enterobacterales	4 (17.39)
**Preferred targeted use of Cefiderocol**	**Isolates**	**N (%)**
Total (23/23)	Kp VIM or NDM +	3 (13.04)
	*P. aeruginosa* MDR	2 (8.7)
	*A. baumannii* MDR	20 (86.96)
	*S. maltophilia*	1 (4.35)
	Other Enterobacterales	0 (0)
**Preferred targeted use of M/V**	**Isolates**	**N (%)**
Total (23/23)	Kp KPC+	19 (82.61)
	*P. aeruginosa* MDR	4 (17.39)
	Other Enterobacterales	3 (13.04)
**Cefiderocol combination therapy against CRAB**	**Molecules**	**N (%)**
Total (23/23)	Rifampicin	3 (13.04)
	Fosfomycin	16 (69.57)
	Polymixin	12 (52.17)
	Ampicillin/sulbactam	9 (39.13)
	Tigecyclin	6 (26.09)
	Never in combination	1 (4.35)
**Cefiderocol combination therapy against Pseudmonas MDR**	**Molecules**	**N (%)**
Total (23/23)	Aminoglycosides	8 (34.78)
	Fosfomycin	16 (69.57)
	Polymixin	8 (34.78)
	Never in combination	2 (8.70)
**Cefiderocol combination therapy against NDM/VIM +**	**Molecules**	**N (%)**
Total (23/23)	Aztreonam	7 (30.43)
	Fosfomycin	10 (43.48)
	Polymixin	7 (30.43)
	Aminoglycosides	5 (21.74)
	Tigecyclin	3 (13.04)
	Never in combination	4 (17.39)
**Cefiderocol combination therapy against KPC and OXA-48 +**	**Molecules**	**N (%)**
Total (23/23)	Fosfomycin	15 (65.22)
	Polymixin	9 (39.13)
	Aminoglycosides	7 (30.43)
	Tigecyclin	3 (13.04)
	Never in combination	4 (17.39)
**M/V combination therapy against KPC +**	**Molecules**	**N (%)**
Total (23/23)	Fosfomycin	13 (56.52)
	Polymixin	6 (26.09)
	Aminoglycosides	9 (39.13)
	Tigecyclin	4 (17.39)
	Never in combination	6 (26.09)

Abbreviations: M/V: meropenem/vaborbactam; KPC: *K. pneumoniae* Carbapenemases; NDM: New-Dehli Metallo-Beta-Lactamases; VIM: Verona Integron-Encoded Beta-Lactamases; MDR: multi-drug resistant; CRAB: Carbapenem-Resistant *A. baumannii*.

## Data Availability

Not applicable.
